# Draft Genome Sequence of the Deep-Sea Basidiomycetous Yeast *Cryptococcus* sp. Strain Mo29 Reveals Its Biotechnological Potential

**DOI:** 10.1128/genomeA.00461-16

**Published:** 2016-07-07

**Authors:** Vanessa Rédou, Abhishek Kumar, Matthieu Hainaut, Bernard Henrissat, Eric Record, Georges Barbier, Gaëtan Burgaud

**Affiliations:** a Université de Brest, Laboratoire Universitaire de Biodiversité et Ecologie Microbienne, Technopôle de Brest Iroise, Plouzané, France; b Division of Molecular Genetic Epidemiology, German Cancer Research Center (DKFZ), Heidelberg, Germany; c Architecture et Fonction des Macromolécules Biologiques, Centre national de la recherche scientifique, Aix-Marseille Université, Marseille, France; d Institut national de recherche agronomique, Marseille, France; e Department of Biological Sciences, King Abdulaziz University, Jeddah, Saudi Arabia; f Institut national de recherche agronomique, Biodiversité et Biotechnologie Fongiques, Aix-Marseille Université, Polytech Marseille, Marseille, France; g Aix-Marseille Université, Biodiversité et Biotechnologie Fongiques, Polytech Marseille, Marseille, France

## Abstract

*Cryptococcus* sp. strain Mo29 was isolated from the Rainbow hydrothermal site on the Mid-Atlantic Ridge. Here, we present the draft genome sequence of this basidiomycetous yeast strain, which has highlighted its biotechnological potential as revealed by the presence of genes involved in the synthesis of secondary metabolites and biotechnologically important enzymes.

## GENOME ANNOUNCEMENT

Yeasts of the *Cryptococcus* genus are widely distributed in the biosphere, ranging from terrestrial to marine habitats (1). Sequence tag-based analysis of deep-sea microeukaryotes occurring in deep-sea habitats revealed *Cryptococcus* as highly abundant taxa in cold methane seep (2), the deep-sea floor (3, 4), and the deep subseafloor (5, 6). A recent RNA-based approach suggested that this phylotype was also metabolically active in deep sedimentary ecosystems (5).

Here, we describe the draft genome sequence of *Cryptococcus* sp. strain Mo29 (= UBOCC 208024), a putative producer of original bioactive compounds. High-quality genomic DNA was extracted using the hexadecyltrimethylammonium bromide (CTAB) and Genomic-tip (Qiagen) methods. Genomic DNA of *Cryptococcus* sp. Mo29 was used to generate shotgun and mate-pair libraries with insert sizes of approximately 350 bp and 8 kb, respectively. A shotgun library was made using the TruSeq DNA PCR-free sample preparation kit and a gel-plus mate-paired-end library was generated with the Nextera mate-pair sample preparation kit. Genome sequencing was performed using Illumina HiSeq 2500 sequencing technologies.

The shotgun library produced 41,481,610 reads. The mate-pair library produced 15,799,276 reads. After quality filtering of reads with more than 90% of bases with base quality greater than or equal to Q20, a total of 28,246,332 shotgun reads (2,85248 Mb) and 7,870,684 mate-pair reads (732 Mb) were retained. The ALLPATHS-LG whole-genome shotgun assembler (7) was used for the creation of the *de novo* genome assembly from these short reads. The assembly contained a total of 174 scaffolds with an average read length of 144,951 bp. The *N*_50_ was 820 kb, and the maximum contig length was 1,690 kb. The total sequence length of the resulting draft genome was 27,528,793 bp with an overall GC content of 50.09%. A total of 174,473 bp were repeats, which constituted 0.63% of the assembled genome size as predicted by repeat masker tool (http://www.repeatmasker.org/). There are 10,009 genes in this fungal genome, as predicted by Augustus 3.0 (8). Our BLAST2GO-based annotation analyses (9) have revealed 57.5% annotated genes, while 42.5% remained unannotated.

The genome analysis of secondary metabolite biosynthesis gene clusters using antiSMASH 3.0 software (10) highlighted the presence of four biosynthetic gene clusters, which included one type III polyketide synthase and one terpene synthase and two clusters that were unknown (also called as others in AntiSMASH annotation). Analysis of the sequence with the CAZy database (11) identified 860 genes with activity involving carbohydrates, including 396 glycoside hydrolases, 130 glycosyltransferases, 31 polysaccharide lyases, 63 carbohydrate esterases, 99 carbohydrate-binding modules, and 141 auxiliary activities. Particularly noteworthy is the finding that *Crypotococcus* sp. Mo29 is equipped with all necessary enzymes for the deconstruction of terrestrial plant cell walls, including cellulases, xylanases, pectinases and ligninases. The finding of numerous genes associated with synthesis of secondary metabolites and CAZyme in the genome sequence of *Cryptococcus* sp. Mo29 suggests that further investigation will result in the discovery of useful gene products that may be exploited for biotechnological application.

### Nucleotide sequence accession numbers.

The nucleotide sequence of the *Cryptococcus* sp. UBOCC 208024 (Mo29) genome is deposited in DDBJ/EMBL/GenBank under accession numbers FKKD01000001 to FKKD01000687. This paper describes the first version of the genome.
